# Ovarian morphology is associated with insulin resistance in women with polycystic ovary syndrome: a cross sectional study

**DOI:** 10.1186/s40738-017-0035-z

**Published:** 2017-05-30

**Authors:** Sara Pittenger Reid, Chia-Ning Kao, Lauri Pasch, Kanade Shinkai, Marcelle I. Cedars, Heather G. Huddleston

**Affiliations:** 1 Center for Reproductive Health, University of California at San Francisco, 2356 Sutter Street, San Francisco, 94115 CA USA; 2 Dermatology, University of California at San Francisco, 1701 Divisadero, San Francisco, 94115 CA USA

**Keywords:** Polycystic ovary syndrome, Ovarian volume, Insulin resistance

## Abstract

**Background:**

Polycystic ovary syndrome (PCOS) is a very common disorder well known to be associated with insulin resistance and metabolic disease. Insulin resistance is likely involved in the promotion of the PCOS reproductive phenotype and may mediate some of the ovarian morphology seen in the disorder. The phenotype of each individual woman with PCOS can vary widely as can her metabolic risk.

**Methods:**

This is a cross-sectional study of patients seen in a multidisciplinary PCOS clinic at the University of California at San Francisco between 2006 and 2014.

All participants underwent systematic evaluation with anthropometric measurements, comprehensive skin exam, transvaginal ultrasound and laboratory studies at the time of their initial visit to the clinic. Serum samples were stored and androgen studies were carried out on all stored samples at the University of Virginia. Logistic regression was employed to evaluate the association between ovarian volume or follicle number and metabolic parameters (fasting insulin, HOMA-IR, fasting glucose, 2 h glucose, waist circumference) and hyperandrogenism (free testosterone, total testosterone, DHEAS, acanthosis nigricans), controlling for age.

**Results:**

Three-hundred thirteen patients seen during the study period met Rotterdam criteria for PCOS and had sufficient measurements for inclusion in our analysis. The odds ratio of elevated HOMA-IR for patients with a maximum ovarian volume >10 cc was 1.9 compared to those with a maximum ovarian volume of ≤10 cc (95% CI 1.0–3.4). The odds ratio of abnormal fasting insulin for patients with higher ovarian volume was 1.8 (95% CI 1.0–3.4) compared with those with lower ovarian volume. Follicle number was not significantly associated with any metabolic parameters.

**Conclusions:**

Increased ovarian volume is associated with markers of insulin resistance in PCOS. In concordance with prior studies, we did not find follicle number to be predictive of metabolic risk. Ovarian volume may serve as a useful tool to aid clinicians in their risk stratification and counseling of patients with PCOS.

## Background

Polycystic ovary syndrome (PCOS) is a very common and heterogeneous disorder. Depending on the criteria used to diagnose PCOS, incidence estimates range from 4–9% of reproductive age women using the 1990 National Institutes of Health criteria [1–3] and as high as 12–21% using the 2003 Rotterdam criteria [4–7]. Among these women, there is a wide range of physical, endocrine and metabolic phenotypes.

It is well known that women with PCOS have an increased risk of insulin resistance, diabetes and metabolic syndrome [8–11]. These metabolic abnormalities are associated with significant long-term morbidity. Insulin resistance, one of the earliest manifestations of metabolic disease, has been demonstrated to contribute significantly to the risk for coronary heart disease (CHD). In a recent National Health and Nutrition Examination Survey (NHANES) study, insulin resistance was shown to be a stronger risk factor for CHD than diabetes [12]. It is well accepted that insulin resistance is a major driver of the metabolic phenotype in PCOS and is compounded by obesity [10, 8]. Insulin resistance likely also plays a role in promoting the PCOS reproductive phenotype as insulin has been shown to increase theca androgen production and may mediate some of the ovarian morphology characteristics commonly seen in PCOS by driving theca cell proliferation [13].

The theca cells in women with PCOS appear to be more responsive to the actions of insulin than those of controls [10]. They secrete more androgens both basally and in response to stimulation by leutinizing hormone (LH) and insulin. Insulin acts as a co-gonadotropin in this case, modulating ovarian steroidogenesis [14].

Screening tests and medical treatments exist to detect and treat those with metabolic disease, but to date, identifying which women with PCOS are at greatest risk has not been straightforward. It would be clinically useful to be able to predict an individual woman’s metabolic risk using her clinical phenotype.

There has been much discussion and disagreement surrounding the specific thresholds defining the ovarian criteria for PCOS. The 2003 Rotterdam criteria are currently the most commonly used standard for classifying the morphology of polycystic ovaries [7]. Under Rotterdam, polycystic ovary morphology (PCOM) is defined as a follicle number per ovary of ≥ 12 and/or an ovarian volume of >10 cc in at least one ovary. The 2014 Androgen Excess and PCOS Society task force recommended the use of ≥ 25 follicles and/or a volume of >10 cc [15]. Because of the existence of these morphologic criteria, specific ultrasound data regarding follicle number and volume exist for most women diagnosed with PCOS, providing clinicians with physical markers that may potentially be useful in stratifying patient phenotype and assessing future risk.

PCOM can be identified in approximately 30% of reproductive aged women [16, 17]. In normovulatory women, this finding has not been associated with an increased risk of metabolic disease [18]. In women with PCOS, the implications of PCOM are not entirely clear. In a very small study of 10 women with PCOS, women with PCOM showed slower glucose disappearance than those without PCOM [19]. A subsequent larger study in 240 Italian women failed to show a difference in insulin in women with and without PCOM. This study, however, used a more liberal definition of PCOM of >9 follicles or a volume >7.5 cc [20]. Other studies on this question group follicle number per ovary and ovarian volume into one phenotype for comparisons and use a variety of definitions of PCOM making comparisons difficult [21]. Accordingly, there is no clear data regarding the associations of the individual components of PCOM and metabolic risk.

We designed a study to determine if ovarian volume (OV) and/or follicle number (FN) are independently associated with abnormal metabolic findings in women diagnosed with PCOS. We hypothesized that ovarian morphology but not follicle number would be associated with a significantly higher odds of hyperinsulinemia among women with PCOS.

## Methods

### Study population and recruitment

This is a prospective, cross sectional study in which patients attending the monthly multi-disciplinary PCOS clinic at the University of California at San Francisco (UCSF) were recruited consecutively between 2006 and 2014. Patients are referred to this clinic for evaluation to determine whether or not they meet criteria for a diagnosis of PCOS and for management recommendations. The clinic is specifically targeted toward women who are not currently seeking fertility treatment. Patients were voluntarily consented to participate in the study based on a research protocol approved by the UCSF Committee on Human Research. Participants were included if they met Rotterdam criteria for PCOS, with two out of three of the following features: oligo- or an-ovulation, clinical and/or biochemical hyperandrogenism, and the presence of 12 or more antral follicles in one ovary and/or a maximum ovarian volume >10 cc [7]. Participants were excluded if they did not have complete ovarian morphology measurements.

### Data collection

All participants presented for a single visit in which they completed a self-administered questionnaire and underwent systematic evaluation with anthropometric measurements, comprehensive dermatologist’s exam, transvaginal ultrasound, and blood work.

### Outcome variables

Outcome variables studied included the following metabolic and androgen measurements. Metabolic: fasting insulin >19 μIU/mL, HOMA-IR greater than 4 [22], fasting glucose >100 mg/dL, 2 h glucose >140 mg/dL, waist circumference >89 cm, and the presence or absence of acanthosis nigricans. Hyperandrogenism: total testosterone >6.8 pg/mL, free testosterone >53 ng/dL, abnormal DHEAS with cutoff for abnormal level determined by the references for each individual performing lab, mFG ≥8, presence of severe acne, and presence of androgenic alopecia.

Participants were asked to abstain from use of oral contraceptive pills and spironolactone for at least 1 month prior to the planned clinic visit. They were also asked to refrain from any type of hair removal, including waxing, plucking or shaving, for 1 month prior to the clinic visit.

A dermatologist performed a comprehensive skin examination of each patient to assess for cutaneous manifestations of hyperandrogenism and hyperinsulinemia. Hirsutism was reported by a modified Ferriman-Gallwey (mFG), A score ≥8 was considered abnormal. Patients were evaluated for the presence of other cutaneous findings of PCOS, including acne, androgenic alopecia, and acanthosis nigricans [7, 23, 24]. Acne was categorized as “present” if the dermatologist determined that treatment for acne would be appropriate. Physiologic acne that was rare and would not be treated was considered “absent”.

Metabolic testing included a 75 g 2-h oral glucose tolerance test. The homeostasis model assessment-estimated insulin resistance (HOMA-IR) was calculated by multiplying fasting plasma insulin μIU/mL (FPI) by fasting plasma glucose mg/dL (FPG), then dividing by the constant 405 as described by Matthews [25]. Assay specifics are not reported for the metabolic parameters as they were drawn and analyzed at multiple different labs according to patient insurance coverage.

Serum was collected and stored for all subjects who consented for participation in our biobank. In April 2014 in preparation for our androgen subanalysis, the serum of all consented subjects was analyzed for total testosterone and sex hormone biding globulin (SHBG). Total testosterone and SHBG were measured in singlet at the University of Virginia (UVA) Center for Research and Reproduction Ligand Assay and Analysis Core Laboratory (Charlottesville VA) and free testosterone was calculated using the law of mass action as previously described [26, 27].

### Exposure variables

Our exposure variables were ovarian volume and follicle number. OV ≤10 cc was considered normal and OV >10 cc was considered elevated. FN was examined at 2 different cutoffs, FN <12 or ≥12; FN <25 or ≥25.

The larger of the two OV and higher FN were utilized for analysis. If one ovary was not measurable due to surgical absence or the presence of a cyst, the measurements of the other ovary were used. If neither ovary could be adequately visualized or if the measurements were absent in the records, the patient was excluded from the analysis.

Each patient’s transvaginal ultrasound was performed with one of two attending reproductive endocrinologists (M.I.C., H.G.H.). A Shizmadzu SDU-450XL machine with a variable 4- to 8-mHz vaginal transducer was used to measure the transverse, longitudinal, and anteroposterior diameters of each ovary to calculate OV using the equation for volume of an ellipse. Follicles between 2 and 9 mm in diameter were counted in each ovary to give the FN.

### Covariates

The covariates measured were age and BMI.

### Statistical analysis

For analyses in which OV and FN were treated as dichotomous variables, Rotterdam criteria or Androgen Excess and PCOS Society criteria for polycystic morphology were used to define the variable as normal or abnormal (OV ≤10 cc or >10 cc; FN <12 or ≥12; FN <25 or ≥25) [7]. T-tests were used to compare means of clinical variables by OV and FN as dichotomous variables. Logistic regression was used to evaluate the effect of OV and FN as dichotomous and continuous variables on metabolic parameters while controlling for age. The number of observations included for each variable of interest is shown in Tables 1, 2, and 3. All computations were performed using SAS 9.3 for Windows, 32-bit edition.

**Table 1:** Cohort Characteristics

	Mean (SD)	Age	BMI
≤10 cc *N* = 151	6.64 (2.0)	28.78 (5.9)	28.43 (7.2)^a^
>10 cc *N* = 162	14.11 (5.7)	28.20 (6.0)	30.33 (7.9)^a^
<12 follicles *N* = 34	8.88 (2.4)	29.00 (8.3)	29.73 (7.6)
≥12 follicles *N* = 279	22.42 (9.7)	28.42 (5.7)	29.38 (7.7)
<25 follicles *N* = 86	16.11 (4.5)	28.74 (6.2)	29.81 (7.8)
≥25 follicles *N* = 227	33.7 (9.6)	27.78 (5.4)	28.37 (6.9)

i. For all comparisons, the largest available ovarian volume and single ovary follicle number was used for each subject

^a^Indicates a significant difference in BMI between subjects with a maximum ovarian volume ≤ 10 cc compared to those with a maximum volume > 10 cc, *p* = 0.03

**Table 2:** Logistic Regression Analysis of Follicle Number and Ovarian Volume as Dichotomous Variables

	Follicle Number ≥12 vs <12	Follicle Number ≥25 vs <25	Ovarian Volume >10 cc vs ≤ 10 cc
	AOR (95%CI)	AOR (95%CI)	AOR (95%CI)
Fasting Insulin >19 μIU/mL	2.2 (0.7–7.0)	0.9 (0.5–1.8)	1.8 (1.0–3.4)
HOMA-IR >4	1.2 (0.4–3.4)	0.8 (0.4–1.6)	1.9 ^a^ (1.0–3.6)
Fasting Glucose >100 mg/dL	0.9 (0.3–2.9)	0.5 (0.2–1.4)	1.8 (0.8–4.0)
2 h Glucose >140 mg/dL	0.9 (0.3–2.9)	0.4 (0.1–1.0)	1.3 (0.6–2.7)
Waist Circ >89 cm	0.9 (0.4–2.2)	0.7 (0.4–1.3)	1.6 (0.9–2.6)
Free Testosterone >6.8 pg/mL	4.2 (0.5–33.2)	2.2 (1.0–4.9)	1.8 (0.8–4.0)
Total Testosterone >53 ng/dL	0.9 (0.2–3.6)	1.5 (0.6–3.3)	1.1 (0.5–2.6)
Abnormal DHEAS	1.1 (0.4–3.0)	1.0 (0.5–2.1)	0.7 (0.4–1.3)
Acanthosis Nigricans Present	0.7 (0.3–1.5)	1.0 (0.6–1.6)	1.4 (0.9–2.3)

i. The largest available ovarian volume and single ovary follicle number was used

ii. Adjusted OR were controlled for age

iii. DHEAS measurements were performed at multiple laboratory sites, the reference cutoff for each site was used to determine normal/abnormal status

iv. HOMA-IR cutoff chosen to reflect more significant insulin resistance [22]

v. Fasting Insulin cutoff chosen to reflect the reference range of the laboratory used by majority of patients in the study

^a^ indicates *p* < 0.05

**Table 3:** Logistic Regression Analysis of Follicle Number as a Continuous Variable

	AOR (95% CI)	*p*
Fasting Insulin >19 μIU/mL	1.00 (0.97–1.03)	0.81
HOMA-IR >4	1.01 (0.98–1.04)	0.65
Fasting Glucose >100 mg/dL	0.98 (0.93–1.02)	0.30
2 h Glucose >140 mg/dL	0.97 (0.92–1.01)	0.16
Waist Circ >89 cm	0.98 (0.95–1.00)	0.07

i. Adjusted OR were controlled for age

### Assays

Testosterone was measured by radioimmunoassay (RIA) [Coat-a-Count Kit; Siemens Healthcare Diagnositics; assay sensitivity 0.2–180 nmol/L; intraassay coefficient of variation (CV) = 4.4%; interassay CV = 6.4%]. SHBG was measured by Immulite [L2KSH2 Kit; Siemens Healthcare Diagnostics; assay sensitivity 6.1–1500.0 ng/dl; intraassay (CV) = 2.8%; interassay CV = 6.5%]. Free testosterone was calculated using the following equation derived from the law of mass action:

$$ \left[\mathrm{fT}\right] = \Big(\left[\mathrm{T}\right]\ \hbox{--}\ \left(\mathrm{N} \times \left[\mathrm{fT}\right]\right)\ /\ \left\{{\mathrm{k}}_{\mathrm{sT}}\left(\left[{\mathrm{C}}_{\mathrm{SHBG}}\right]\ \hbox{--}\ \left[\mathrm{T}\right] + \mathrm{N}\left[\mathrm{fT}\right]\right)\right\} $$

where k_sT_ = affinity constant of SHBG for T, N = k_aT_Ca +1, k_aT_ = affinity constant of albumin for T, and Ca = albumin concentration, assumed to be 4.5 gm/dL [26, 27].

## Results

### Study population

Four-hundred thirty-nine patients seen in the PCOS clinic during the study time period consented for inclusion in the study. Of those, 355 were confirmed to have met Rotterdam criteria for PCOS. Fourty-two subjects meeting Rotterdam criteria were excluded because they did not have complete ultrasound data. Three-hundred thirteen patients meeting Rotterdam criteria for PCOS and having sufficient ovarian measurements for analysis comprised the study population.

Of the included patients, 89% met the Rotterdam FN criteria for PCO and 52% met the OV criteria (Table 1). Thirty-four patients had a FN <12 (8.88 ± 2.39), 279 patients had a FN ≥12 (22.42 ± 9.69). One-hundred fifty-one patients had an OV of ≤10 cc (6.64 ± 1.97), 162 patients had an OV >10 cc (14.11 ± 5.73). Two-hundred twenty-seven patients had a FN <25 (16.11 ± 4.5) and 86 had a FN ≥25 (33.7 ± 9.6). All groups were similar with respect to age, but patients with OV >10 cc had higher mean BMI, waist circumference, fasting insulin and HOMA-IR (Tables 1 and 2).

### Follicle number

The analysis of FN as a dichotomous variable, controlling for age, indicates that FN as defined by Rotterdam criteria does not predict abnormal metabolic indices among PCOS women in our study (Table 2). We performed an additional regression analysis examining FN as a continuous variable to confirm that the predetermined cut offs were not artificially concealing a difference. There was no significant relationship between FN as a continuous variable and any of the metabolic indices (Table 3).

### Ovarian volume

The logistic regression analysis of OV as a dichotomous variable, controlling for age, indicates that the odds of an abnormal fasting insulin was 1.8 and the odds of an abnormal HOMA- IR 1.9 for patients with a maximum OV >10 cc compared with patients with a maximum ovarian volume ≤10 cc. The relationship of OV with fasting glucose and 2-h glucose was not significant (Table 2).

Controlling for BMI attenuated the relationship between insulin resistance and OV such that it was no longer significant (data not shown). However, BMI and insulin resistance are highly co-linear. Further physiologic evidence suggests that insulin resistance may be on the causal pathway linking BMI and OV due to the known stimulatory effect of insulin on ovarian thecal cells [13]. For these reasons, a decision was made not to use BMI in the final model.

We also examined the relationship between ovarian features and abnormal waist circumference. Similar to the other markers of insulin resistance, OV was related to increased waist circumference (although this finding did not reach significance) while FN was not. The OR of having a waist circumference >89 cm in patients with and OV >10 cc was 1.6 (0.9–2.6, *p* 0.08) compared to patients with an OV ≤10 cc. For FN ≥12 vs <12 the unadjusted OR was 0.9 (95% CI 0.4–2.2, *p* 0.9).

### PCO morphology and hyperandrogenism

In our subgroup analysis of the 150 participants with banked serum (48% of all participants), we found no association of OV with elevated free or total testosterone. FN when treated as a dichotomous variable of ≥12 vs <12 was also not associated with hyperandrogenemia. When FN ≥25 vs <25 was examined, however, a FN ≥25 was positively associated with free testosterone >6.8 pg/mL (Table 2).

## Discussion

PCOS is a common endocrinopathy with a highly heterogeneous presentation. The association of PCOS with insulin resistance is well-known, however there is a lack of understanding regarding which patients with PCOS are most at risk of suffering adverse metabolic consequences. We investigated whether transvaginal ultrasound findings can be used to identify women with concurrent abnormal metabolic phenotype.

In our cohort, women with PCOS and OV > 10 cc were 2 times more likely than those with OV ≤ 10 cc to exhibit biochemical markers of insulin resistance. Conversely, we found that although the vast majority of patients with PCOS meet the FN criteria (>12 follicles per ovary), this finding is not associated with any abnormal metabolic parameters. Similarly, subjects meeting the newly recommended FN cutoff of 25 did not display more metabolic abnormalities than those not meeting this criteria. In summary, ovarian volume, but not follicle number, appears to predict metabolic abnormalities in a population of women with PCOS.

To date, there has been limited investigation regarding the individual components of PCO morphology and metabolic disease in adult women. One study examining the relationship of FN and OV with androgen levels and metabolic markers in women with PCOS (*n* = 88) failed to identify a correlation between OV or FN and indices of insulin resistance [28]. However, other smaller reports have suggested a relationship. In a study of 50 women with PCOS, Carmina showed that OV correlates with serum insulin (*r* = .37, *P* < .01) and the Quicki (*r* = −.31. *P* < .05) but does not correlate with BMI, LH, FSH or circulating androgens [29]. Among adolescents, Villa compared ovarian volume between 86 girls with PCOS and 48 controls, demonstrating that OV was associated with circulating insulin levels and markers of insulin resistance [30]. To our knowledge, our study is the largest study of women with PCOS to report the association between ovarian volume and markers of insulin resistance.

While there are multiple proposed explanations surrounding the pathophysiology of PCOS, it is well accepted that insulin resistance is a major driver of the metabolic phenotype and is compounded by obesity [8, 10]. Insulin resistance is likely also a key contributor to the PCOS reproductive phenotype. Systemic hyperinsulinemia due to peripheral insulin resistance provides a mitogenic stimulation of ovarian theca cells leading to an expansion of the theca cell compartment and increased androgen production [31–33]. Additionally, insulin has been shown to lower sex hormone binding globulin, which can lead to increased free androgen levels and clinical hyperandrogenism. In turn, biochemical hyperandrogenemia likely exacerbates peripheral insulin resistance [10]. The insulin-mediated theca cell expansion likely leads to increased stromal volume within the ovary. Our data reinforce the idea that increased OV may be serving as a measurable biomarker of increased levels of circulating insulin.

It is important to note that this cohort consisted exclusively of women with a confirmed diagnosis of PCOS. While some prior studies of ovulatory women meeting PCO morphology criteria have indicated an increased risk of higher fasting insulin and possibly insulin resistance [34], the majority of the data has not demonstrated an increased risk of metabolic dysfunction in regularly cycling women who meet only the follicle number or volume criteria for PCO [17, 34–37]. It is possible that the ovaries of women without PCOS who meet ovarian volume criteria are enlarged due to increased follicular mass rather than the increased theca cell/stromal mass possibly explaining the lack of association between ovarian volume and metabolic findings in a non-PCOS population. To date, there are no recommendations for ovulatory women meeting PCO criteria to undergo metabolic screening beyond the routine for their age and clinical risk factors.

Also important to consider is that cutoff for HOMA-IR in our study was 4, which is higher than in some similar studies. After many population based studies, there is no single set value for HOMA-IR that is consistently used to define insulin resistance [22]. In determining which value we would use, we intentionally selected a value intended to identify more significant cases of insulin resistance.

Our study is strengthened by the systematic way in which all patients were examined and diagnosed under the supervision of one of two attending physicians. The clinic structure and data collection methodology remained unchanged over the time that subjects were recruited. Further, our ability to use banked serum to test all subjects’ testosterone concentrations at a single laboratory using a single, well-validated RIA technique strengthens our findings related to hyperandrogenemia. Our study is potentially limited by a lack of heterogeneity in FN, skewed heavily towards numbers exceeding the Rotterdam cut off of 12. It is also important to note that Rotterdam FN criteria were used in diagnosing our patients with PCOS, which may be overly inclusive in light of current ultrasound technology and new guidelines recommending higher FN cutoffs. Finally, our sample size was insufficient to conclusively determine if OV has an effect on metabolic factors independent of BMI.

## Conclusions

In summary, among the PCOS women in our study with an OV >10 cc the odds ratio for abnormal biochemical markers of insulin resistance was two times that of women with normal OV, indicating that OV is an important factor associated with metabolic risk in women with PCOS. In contrast, follicle number was not associated with clinical evidence of insulin resistance. Ovarian volume may thus serve as a physical biomarker of systemic hyperinsulinemia and the measurement of OV may provide a useful tool to aid clinicians in their risk stratification and counseling of patients with PCOS.

## Abbreviations

BMI: Body mass index
CHR: Committee on Human Research
FN: Follicle number
FPG: Fasting plasma glucose
FPI: Fasting plasma insulin
HOMA-IR: Homeostasis model assessment-estimated insulin resistance
LH: Luteinizing hormone
mFG: Modified Ferriman-Gallwey
NHANES: National Health and Nutrition Examination Survey
OR: Odds ratio
OV: Ovarian volume
PCOM: Polycystic ovary morphology
PCOS: Polycystic ovary syndrome
RIA: Radioimmunoassay
SHBG: Sex hormone binding globulin
UCSF: University of California, San Francisco
UVA: University of Virginia
